# Sphingolipid abnormalities in encephalomyeloradiculoneuropathy (EMRN) are associated with an anti‐neutral glycolipid antibody

**DOI:** 10.1002/2211-5463.13578

**Published:** 2023-03-02

**Authors:** Tatsuro Mutoh, Akihiro Ueda, Yoshiki Niimi

**Affiliations:** ^1^ Department of Neurology and Neuroscience Fujita Health University Hospital Toyoake Japan; ^2^ Fujita Health University Central Japan International Airport Clinic Tokomane Japan

**Keywords:** anti‐neutral glycolipid antibody, encephalitis, sphingolipid, encephalomyeloradiculoneuropathy (EMRN), innate immunity, neuroinflammation

## Abstract

Accumulating evidence suggests that various sphingolipids and glycosphingolipids can act as mediators for inflammation or signaling molecules in the nervous system. In this article, we explore the molecular basis of a new neuroinflammatory disorder called encephalomyeloradiculoneuropathy (EMRN), which affects the brain, spinal cord, and peripheral nerves; in particular, we discuss whether glycolipid and sphingolipid dysmetabolism is present in patients with this disorder. This review will focus on the pathognomonic significance of sphingolipid and glycolipid dysmetabolism for the development of EMRN and the possible involvement of inflammation in the nervous system.

## Graphical abstract

This review illustrates sphingolipid and glycolipid abnormalities in a new neuroimmunological disorder involving the whole nervous system: encephalomyeloradiculoneuropathy (EMRN). Abnormal production of autoantibodies against neutral glycolipids and sphingolipid dysmetabolism is associated with abnormal innate immunity status in this disorder. EMRN is the prototype of sphingolipid and glycolipid abnormalities leading to the development of neuroinflammation and possibly neurodegeneration in human neurological disorders.

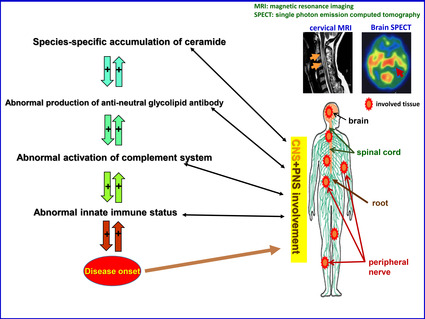

**Abbreviations**
anti‐NGL antibody: anti‐neutral glycolipid antibody
Cer: ceramides
CNS: central nervous system
CSF: cerebrospinal fluid
EMRN: encephalomyeloradiculoneuropathy
GalCer: galactosylceramide
GD: Gaucher disease
GlcCer: glucosylceramide
GSL: glycosphingolipid
iNPH: idiopathic normal pressure hydrocephalus
LacCer: lactosylceramide
LE: limbic encephalitis
nSMase2: neutral sphingomyelinase 2
PD: Parkinson's disease
PNS: peripheral nervous system
PVDF membrane: polyvinylidene difluoride membrane
RP: relapsing polychondritis

Previous studies have revealed evidence that many neurological disorders involve neuroinflammation, which may play important roles in neurodegeneration in such disorders. Heretofore, many glycobiological studies have shown that various sphingolipids and glycolipids can play roles as signaling molecules for neuroinflammation in the brain and peripheral nervous system (PNS) as well [1]. Moreover, recent studies have also indicated that dysregulation of glycosphingolipid (GSL) metabolism appears to induce neurodegenerative disorders [2, 3].

The synthesis of ceramide (**Cer**) is the first step of all complex GSL‐synthesizing pathways, and the accumulation of **Cer** can cause neuronal cell death by inducing oxidative stress and proinflammatory cytokine expression [4, 5]. Therefore, it is necessary to keep steady‐state levels of **Cer** in the nervous system for the normal function of neurons. **Cer** constitutes many subspecies exhibiting different acyl chain lengths, hydroxylation, and saturation of both the sphingoid base and fatty acid moieties. More importantly, human brain gray matter is known to contain abundant **Cer** C16 and C18, whereas C24 is abundant in human white matter and myelin [6]. Thus, we should be careful when examining the acyl chain length of **Cer** fatty acid moieties in various human samples such as brain and cerebrospinal fluid (CSF) [6]. Moreover, plasma **Cer** levels can indicate the degree of impairment of the cognitive function. Elevations of **Cer** levels in subjects with mild cognitive impairment might predict further cognitive decline and future hippocampal volume loss [7]. A very recent study has indicated that plasma **Cer** levels are well correlated with the severity of major depressive disorder in humans and animal models [8]. Interestingly, normalization by anticeramide synthase antibody and/or ceramide synthase inhibitor rescued symptoms of major depressive disorder in model animals [8].

So far, sphingolipid and GSL levels in the CSF or brain tissues have not been. available in patients with various neurological disorders [9].

On the other hand, the complement pathway is an important player in innate immune responses and it consists of three different activating pathways, all of which converge with the activation of complement C5, yielding the C5a active fragment and finally leading to the formation of membrane attack complex [10]. Intriguingly, previous studies have shown that the production of anti‐neutral glycolipid (NGL) autoantibody (which leads to the activation complement system) has been observed in experimental and human Gaucher disease (GD) and amyotrophic lateral sclerosis (ALS) transgenic mice ([1, 2, 3, 4, 5, 6, 7, 8, 9, 10, 11, 12, 13], see [14] for review).

## Encephalomyeloradiculoneuropathy

A case report of encephalomyeloradiculoneuropathy (EMRN) was reported by Blennow *et al*. [15] for the first time. They reported four children with good self‐limiting prognosis exhibiting natural complete recovery, with a condition involving both the central nervous system (CNS) and PNS, and exhibiting encephalopathy and neuropathy [15]. This disorder can attack adults as well causing a variety of symptoms such as motor weakness, myelopathy, neuropathy, encephalopathy, and dysautonomia [16, 17]. So far, there are less than 20 reported cases in the literature, and therefore, its pathogenesis remains to be fully elucidated.

**Fig. 1:**
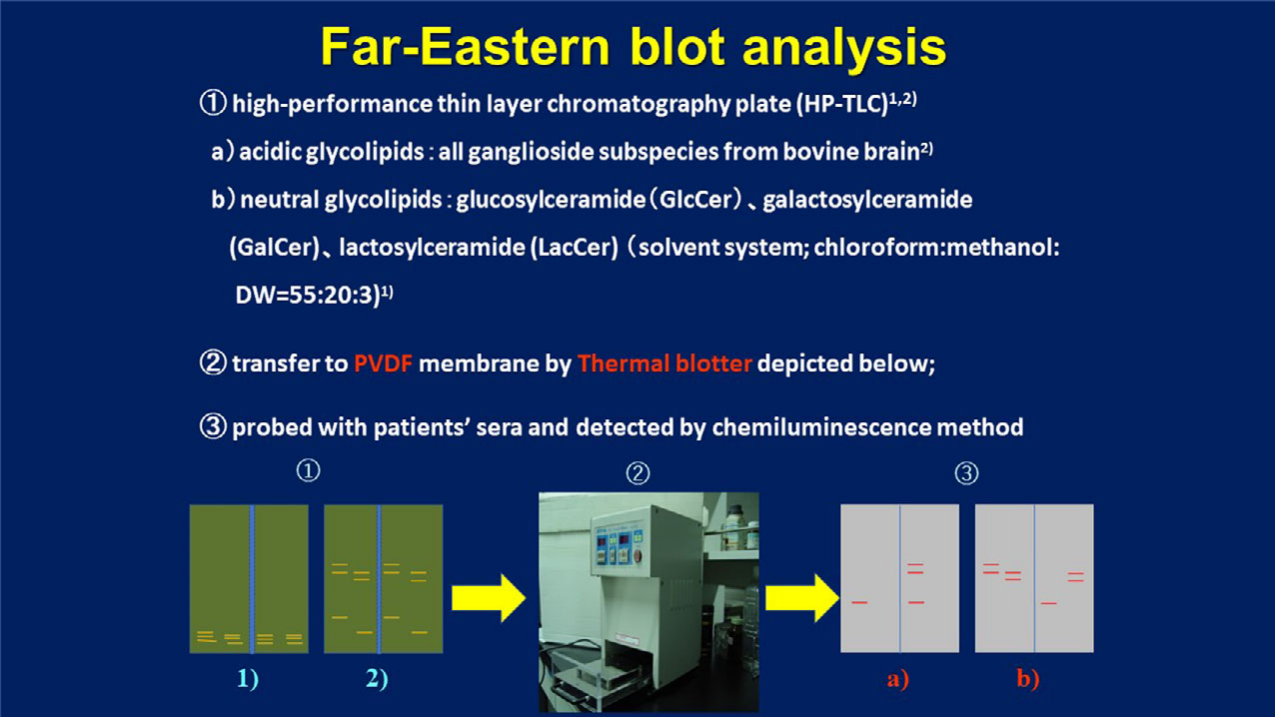
Schematic presentation of how to perform Far‐Eastern blot analysis. Each antigen solution was applied onto high‐performance thin‐layer chromatography plates and developed with the respective organic solvent system. These separated antigens were then transferred onto a PVDF membrane using a thermal blotter (depicted in [2]) and were subjected to regular western blot analysis using patient sera and CSF. Positive bands were detected by chemiluminescence.

In 2014, we encountered four EMRN patients exhibiting a single phase of both CNS and PNS involvement; we detected for the first‐time anti‐NGL autoantibodies, especially anti‐lactosylceramide (LacCer), in sera and CSF from these patients at the acute phase. Surprisingly, we could not detect these antibodies after recovery of the disease following immunomodulatory therapy, indicating that anti‐NGL titers were well correlated with disease status [18]. To detect these anti‐NGL antibodies, we employed the Far‐Eastern blot method, which was originally developed by Taki T *et al*. [19]. This method combined two steps: (a) the separation of sphingolipids and glycolipids with a thin‐layer chromatography plate and (b) blotting these partially purified antigens on a polyvinylidene difluoride (PVDF) membrane. Therefore, we can detect true‐positive bands but not false‐positive bands derived from contaminating lipids in the respective antigen solution (Fig. 1). Thereafter, several patients showed remitting and relapsing clinical courses and incomplete presentation of clinical involvement of either CNS or PNS symptoms in association with anti‐NGL antibodies, although most of these cases exhibited severe autonomic nervous system involvement. These patients also showed altered titers of the autoantibodies against NGLs in relation to their clinical status of the disease [20, 21, 22]. It is very important to examine the pathological states in the brain and PNS in detail to understand the pathogenesis of EMRN.

Therefore, we investigated peripheral innate immune status by measuring peripheral active C5 complement, C5a levels, and sphingolipid and glycolipid levels in CSF obtained from the acute phase of the disease. We compared them with those from neurologically‐normal controls and another neurological disorder, Parkinson's disease (PD) [23]. We observed a significant upregulation of serum C5a levels and a significant accumulation of species‐specific **Cer** in CSF, which suggests that the abnormal activation of innate immunity might play an essential role in the development and/or progression of EMRN [23]. Later, we also confirmed significant upregulation of CSF C5a levels in these patients (T. Mutoh & Y. Niimi, unpublished results). In addition, these data may also open new therapeutic strategies involving the application of anticomplement therapy and/or anticeramide therapy for the treatment of patients who exhibited sometimes the fulminant course of the disease. Unfortunately, no autopsied case of EMRN has been reported. Therefore, currently, the molecular mechanisms for the occurrence of anti‐NGL autoantibodies and disturbed **Cer** metabolism in EMRN remain to be elucidated.

## Relapsing polychondritis with limbic encephalitis

In addition to EMRN, anti‐NGL antibodies, specifically against glucosylceramide (GlcCer), are present in a limited number of patients with relapsing polychondritis (RP), which is complicated by limbic encephalitis (LE). RP is characterized by a chronic multisystem disorder of unknown etiology. RP patients usually exhibit recurrent episodic inflammation of cartilaginous tissues. Inflammation often attacks proteoglycan‐rich structures, such as eyes, inner ears, heart, blood vessels, and kidneys [24]. It is intriguing that sera from patients without LE did not contain anti‐GlcCer antibodies, but patients with LE‐complicated RP were positive for anti‐GlcCer antibodies [25]. Following our previous report, the authors did not detect anti‐NGL antibodies other than anti‐GlcCer antibodies in patients with RP with LE (unpublished results). Recently, as mentioned earlier, Pandey *et al*. [11] detected anti‐GlcCer antibodies in sera from patients with Gaucher disease (GlcCer‐accumulated lysosomal storage disorder).

## GSLs as neuroinflammatory mediators in the nervous system

### Sphingolipid and glycolipid as inflammatory signaling molecules in the nervous system

#### Ceramide

Sphingolipids are distributed in the eukaryotic cell membrane and they work as signaling molecules regulating various cellular processes, such as cell proliferation, growth, survival, immune‐cell trafficking, vascular and epithelial integrity, and inflammation. Sphingolipid alterations, which may include an increase in **Cer**, are observed during neuroinflammatory disease, as discussed previously [7, 8]. Moreover, recent studies of the action of sphingolipid metabolites and new perspectives on their roles in regulating chronic inflammation have been proposed. It was reported that short‐chain ceramide may provoke the expression of anti‐inflammatory cytokines in microglial cells [26], whereas long‐chain fatty acids containing ceramide induce the expression of proinflammatory cytokines in astrocytes [27]. Therefore, we should not ignore the length of the acyl chain of fatty acid moieties of **Cer**. Alterations in membrane microdomains, through the elevation of **Cer** and its tendency to self‐associate, influence interactions of lipids or signaling proteins within the membrane microdomains. A variety of signaling cascades in immune cells such as activation of B cells, bacterial pathogens, and release of cytokines during infection have been reported to be exerted through these ceramide‐rich platforms [28].

Accumulating evidence has suggested that **Cer** levels are significantly upregulated in many neurodegenerative disorders such as PD, dementia of Lewy body disease, and amyotrophic lateral sclerosis [29]. Various neurodegenerative disorders typically showed neuroinflammatory findings. These reports highlighted a tight link between GlcCer and LacCer with the expression of mature proinflammatory cytokines that are necessary for the establishment of chronic neuroinflammation.

Intriguingly, anti‐Cer autoantibodies have been reported in patients with some peripheral neuropathy and leprosy [30, 31]. These anti‐Cer antibodies can recognize C2, C8, C16, C18, C20, and C24 Cer subspecies [32]. Moreover, anti‐Cer antibodies can protect epithelial cells from radiation‐induced gastrointestinal tract syndrome [33].

#### Glucosylceramide

It is well known that gangliosides are particularly distributed in the gray matter and neurons, while galactosylceramide (GalCer) and their sulfated derivative sulfatides are highly enriched in oligodendrocytes and myelin [34]. Most GSLs are derived from GlcCer in the nervous system, whereas some GSLs are derived from GalCer and its sulfated product sulfatides. GD is a lysosomal storage disease caused by a genetic deficiency of glucocerebrosidase 1 (GBA1), resulting in the accumulation of GlcCer and glucosylsphingosine in patients. Moreover, heterozygous mutation of the *GBA1* gene is now known to be a strong risk factor for PD development [35]. Many studies have implicated that the level of GlcCer and **Cer** contents might be closely related to neuroinflammatory findings in the brain from PD patients. Moreover, patients with GD and a model mouse of GD exhibit anti‐GlcCer autoantibodies; these autoantibodies activate the complement system and thereby elicit neuronal destruction and pathological progression of GD patients [11].

#### Galactosylceramide

Galactosylceramide is mainly produced in oligodendrocytes and constitutes myelin in conjunction with another main constituent, sulfatide. The 2‐hydroxylated fatty acid moieties of GalCer are produced only in oligodendrocytes, because its synthesizing enzyme, fatty acid‐2 hydroxylase, is mostly present in these cells [36]. We recently found that patients with idiopathic normal pressure hydrocephalus (iNPH), a syndrome showing Parkinsonism, urinary incontinence, and mental dysfunctions, exhibited a specific and significant reduction in all GalCer subspecies, including 2‐hydroxylated fatty acid‐containing GalCer subspecies, in CSF, compared with neurologically‐normal subjects and PD cases, which suggests that severe dysmetabolism of GalCer may predispose towards this syndrome. At present, the pathogenesis of iNPH is still under investigation. Of note, dysmetabolism of the other NGLs and sphingolipids was not observed in iNPH patients. Although the molecular mechanisms for such a specific and significant reduction in GalCer levels remain to be elucidated, GalCer levels in CSF could serve as an excellent surrogate marker for this disorder [37]. As GalCer is the main lipid of myelin, it therefore might be present in some abnormalities in myelin structure. A previous study has already demonstrated that 2‐hydroxylated fatty acid‐containing GalCer subspecies are necessary to maintain the normal structure of the myelin sheath [36]. Intriguingly, patients with iNPH usually show radiological involvement of white matter in the brain [38].

#### Lactosylceramide

Pannu R *et al*. [39] reported that LacCer regulates lipopolysaccharide/interferon γ‐induced expression of proinflammatory mediators in rat primary astrocyte cultures. In this model animal, silencing of the LacCer synthase gene with antisense oligonucleotides decreased cytokine‐induced inducible nitric oxide synthase (iNOS), TNF‐α, and IL‐1β gene expression, and this was rescued by LacCer supplementation to the animals.

Moreover, it has also recently become apparent that altered GSL metabolism may impair blood–brain barrier function and render the brain susceptible to the infiltration of peripheral myeloid and lymphoid cells, antibodies, and proinflammatory cytokines that would otherwise be restricted from the brain [40]. For example, silencing of the LacCer synthase gene with antisense oligonucleotide in TNF‐α‐ and IFN‐γ‐stimulated astrocytes attenuates the expression of adhesion molecules such as ICAM‐1 and vascular cell adhesion molecules [41].

LacCer itself is closely related to neuroinflammatory responses, as mentioned earlier; dysregulated LacCer metabolism may be involved in EMRN patients, but we have not detected altered LacCer levels in CSF from EMRN patients so far [23]. Our study, however, revealed that the abnormal activation of the complement system (C5 complement) in peripheral and possibly central immune systems leads to the development of abnormal innate immunity status and neuroinflammation in patients with EMRN [23, unpublished results]. Our neuroradiological studies of EMRN patients confirmed the presence of neuroinflammation in these patients [18].

## GSL as inflammatory mediators in the nervous system

### Anti‐neutral glycolipid autoantibodies as another pathway for inflammation in the nervous system

Previous studies have revealed that the production and reaction against the antigens of autoantibodies is one of the immune‐mediated pathways leading to the establishment of neuroinflammation. The first line of evidence was reported in 2004 by Yamaguchi A *et al*. [42]. They clearly disclosed that Sandhoff disease model mice developed anti‐GM2 ganglioside autoantibodies. Moreover, they proved that these autoantibodies are closely related to the development of pathological progression in these mice. The same phenomenon (anti‐GlcCer antibody) was also later reported in 2017 in Gaucher disease patients and a mouse model of this disease [11]. They strongly indicated that anti‐GlcCer autoantibodies cause neuronal dysfunction by killing neurons in these mice. Importantly, before these discoveries, our group already discovered anti‐GlcCer autoantibodies in patients with RP + LE in 2006 and in patients with EMRN in 2014 [18, 25]. Heretofore, all EMRN patients also exhibited anti‐LacCer autoantibodies in their sera and in CSF. As mentioned before, EMRN patients exhibit increased levels of the peripheral active C5 complement (C5a fragment) suggesting that anti‐NGL autoantibodies in these patients might activate innate immunity resulting in the significant upregulation of active complement levels in these patients. At present, it is unknown why these EMRN patients exhibit anti‐NGL antibodies and therefore the detailed molecular mechanisms need to be clarified further, although the aforementioned studies on GM2 gangliosidosis and Gaucher disease suggest that excess amounts of respective glycolipids may be a strong inducer for the development of autoantibodies against these glycolipids in the respective disorders.

Another immunological possibility is the immunological recognition of locally accumulated glycolipids by CD1d molecules and/or other related molecules, although at present CD1d molecules have been reported to be able to recognize α‐ but not β‐GlcCer [43].

**Fig. 2:**
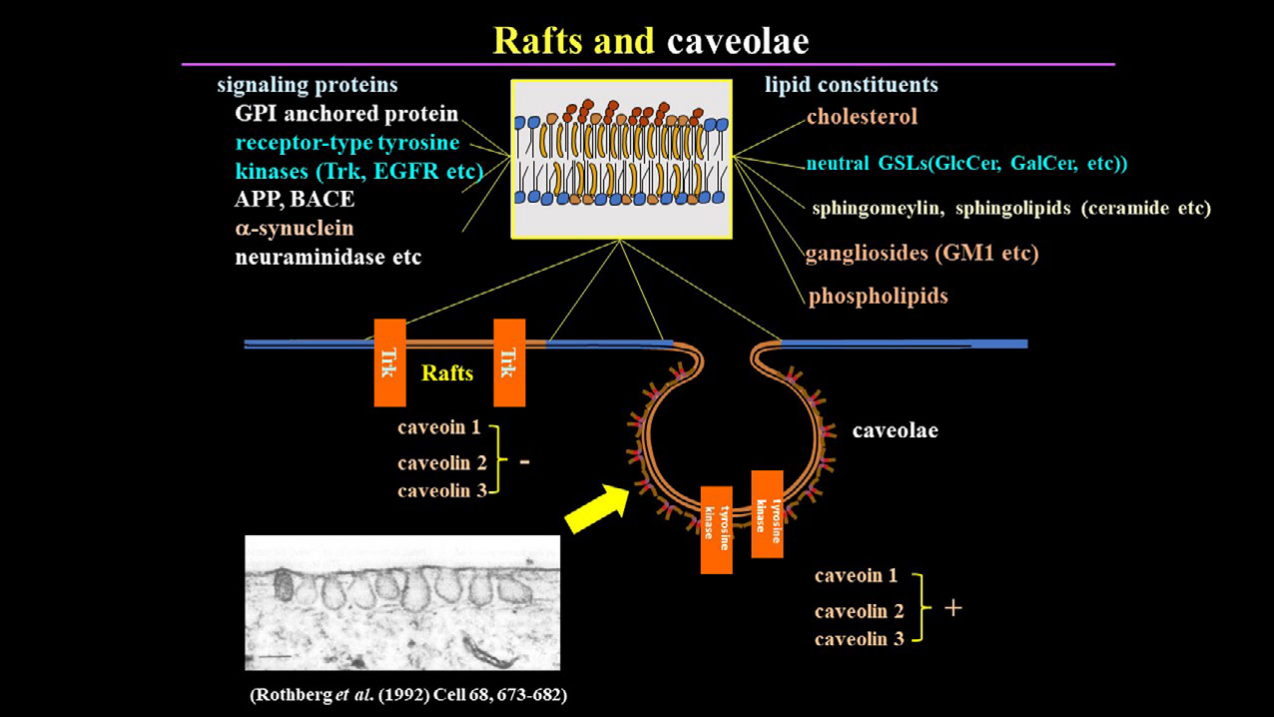
Lipids rafts and caveolae. Lipid raft‐deficient structural proteins such as caveolin and caveolar structure comprise a special membrane microdomain for extracellular communication and signaling in cells. Lipid rafts are usually present in neuronal cells, whereas caveolae are present in dividing cells such as epithelial cells. This special membrane microdomain is formed by molecular interaction of lipids such as cholesterol, sphingolipids, and glycolipids, and protein components such as receptor‐type tyrosine kinases and ion channels, which are recruited to this special domain for efficient signal transduction across the membrane.

More importantly, these anti‐neutral and acidic glycolipid autoantibodies can elicit significant effects on the intracellular signaling pathway of lipid raft‐dependent cellular events [25, 44, 45], and unpublished results. Lipid rafts are the functional membrane microdomain where cholesterol, sphingolipids, glycolipids, signaling molecules (such as receptor‐type tyrosine kinases), and ion channels are concentrated (Fig. 2). Our previous studies have shown that both anti‐GlcCer antibodies and anti‐GM1 ganglioside antibodies provoke the re‐arrangement of Trk‐neurotrophin receptor, high‐affinity nerve growth factor receptor, in PC12 cells [25, 44]. Moreover, anti‐GM1 ganglioside autoantibodies inhibit neutral sphingomyelinase 2 (nSMase2) activity in the membrane fraction via re‐localization of nSMase2 protein from the plasma membrane to the cytosol [45]. These *in vitro* experimental data strongly suggest that anti‐neutral and acidic glycolipid autoantibodies might exert dramatic effects on the membrane‐associated intracellular signaling pathways of neurons and glial cells *in vivo*.

## Conclusion

In summary, glycolipids and sphingolipids can act as a strong inducer of neuroinflammation in various human disorders. The data also suggest that autoantibodies against these glycolipids and sphingolipids also exert profound effects on neuroinflammation and neurodegeneration in humans. Further detailed examinations employing lipidomic and immunological analyses are required for improving our understanding of neurological disorders.

## Author contributions

TM conceived and designed the project, TM, AU, and YN acquired the data, TM and YN analyzed and interpreted the data, AU and YN took care of patients, TM wrote the paper and TM, AU, and YN revised the manuscript.

## Conflict of interest

The authors declare no conflict of interest.
